# Multi-Ancestry Genome-Wide Association Study in All of Us for Primary Open- Angle Glaucoma

**DOI:** 10.21203/rs.3.rs-7754041/v1

**Published:** 2025-10-26

**Authors:** Kiana Tavakoli, Bonnie B. Huang, Tara Mirmira, Nichole Ma, Robert N. Weinreb, Sally L. Baxter

**Affiliations:** University of California, San Diego; University of California, San Diego; University of California, San Diego; University of California, San Diego; University of California, San Diego; University of California, San Diego

**Keywords:** Glaucoma, Open-Angle, Genetic Association Studies, Genetic Loci

## Abstract

This study aims to identify new genetic loci associated with primary open-angle glaucoma (POAG) and explore shared genetic risk factors across African, European, and Admixed American/Latino populations. Genome-wide Association Study (GWAS) utilizing data from the *All of Us* Research Program. The study included 374,254 participants, with 4,305 individuals diagnosed with POAG and 369,949 controls. Participants were categorized by ancestry: European, African, and Admixed American/Latino. We used short-read sequencing data and applied strict quality control measures (MAF > 0.01, INFO > 0.8). GWAS were conducted for each ancestry group using a logistic mixed model, adjusting for age, sex, and the top 11 principal components. A fixed-effect meta-analysis combined the results across ancestries. Genome-wide significance was set at p<5×10^−8^. The primary outcome measures were the identification of genetic loci associated with POAG, and the analysis of transcription factors linked to these loci in relevant tissues. In the European cohort, we identified four novel loci associated with POAG, linked to the *TUT4, RYK, MOXD1, and UBAP2* genes, as well as the previously known *TMCO1* locus. In the African cohort, we found five new loci, including *TSPAN17, SLC16A7, LOC100506869, LINC02388, and LOC107984606*. For the Admixed American/Latino cohort, we identified *GATA5, FAM135B, and LINC00871* genes as novel loci. Our analysis identified three novel loci in individuals of European ancestry, mapped to the genes *TUT4, RYK, and MOXD1*. In addition, five novel loci were detected in the GWAS of African ancestry participants, and four novel loci were identified in individuals of Admixed American/Latino ancestry. These findings indicate that the genetic determinants contributing to POAG may differ across populations, underscoring the importance of accounting for population-specific genetic architectures in the study of complex traits. Given the substantial variation in POAG prevalence among ancestries, it is plausible that certain genetic variants exert ancestry-specific effects. Consequently, conducting ancestry-stratified GWAS is essential for elucidating these unique genetic contributions.

## Introduction

Primary Open Angle Glaucoma (POAG) is the leading cause of irreversible blindness globally.^1^ It is a degenerative disease of the optic nerve that leads to progressive vision loss.^2^ The transferability of genetic findings between populations is understood to be limited by ancestry-specific differences in linkage disequilibrium, minor allele frequency, and potentially differences in causal variants, which pose significant limitations to our understanding of the genetic architecture of POAG in non-European populations. This disparity may result in unequal benefits among different populations from precision medicine, as genetic risk models derived from large-scale studies conducted in European populations exhibit high predictive power in European samples but demonstrate poor predictive accuracy in non-European samples.^3^ Consequently, enhancing ethnic and ancestral diversity among study participants is crucial for identifying understudied mechanisms of disease and ultimately ensuring equitable genetic findings.^4,5^ Specifically, in large studies that focus only on people of European ancestry, disease-critical genetic variants may be missed because they are either rare or completely absent.

In this study, we report a genome-wide association study (GWAS) of POAG utilizing the *All of Us* Research Program dataset, a diverse nationwide database in the United States that emphasizes the recruitment of populations historically underrepresented in biomedical research.^6^ Our analysis includes individuals of European, African, and admixed American/Latino ancestries. We provide a comprehensive discussion on the identification of novel loci associated with POAG and examine the extent to which genetic signals are shared across ancestries, as well as the presence of ancestry-specific genetic signals. Our findings offer valuable insights into the etiology of POAG and underscore the importance of conducting genetic studies within non-European populations.

## Methods

### Study cohort:

Data were sourced from the *All of Us* Research Program, a landmark research initiative aimed at advancing precision medicine by collecting and analyzing health data from diverse populations.^6^ The program encompasses demographic, geographic, and medical diversity, including historically underrepresented populations such as ethnic minorities and individuals from underserved communities. All participants provided written informed consent, demonstrating their voluntary participation in the study and understanding of its purpose. Data sources for the *All of Us* Research Program include electronic health records, physical measurements, surveys, biospecimens, and wearable technology data.Prospective enrollment and data collection were approved by an independent institutional review board, with written informed consent obtained from all participants. The *All of Us* Data Research Center harmonized the data into the Observational Medical Outcomes Partnership (OMOP) common data model, a standardized framework for representing observational health data from diverse sources. To protect participant privacy, the Data Research Center applied measures such as deidentification and date shifting before making the data available on the *All of Us* Researcher Workbench. Secondary analyses of these deidentified datasets were classified as not involving human subjects research by the University of California San Diego Institutional Review Board. This study was conducted in accordance with the Declaration of Helsinki and followed the STROBE (Strengthening the Reporting of Observational Studies in Epidemiology) guidelines for observational research..^7^

### Phenotyping:

There were 403,916 individuals who were enrolled in *All of Us* and had short-read sequencing data available on the *All of Us* Researcher Workbench Controlled Tier dataset version 8. Participants diagnosed with POAG were identified using SNOMED concept ID 77075001 (“Primary open angle glaucoma”) derived from electronic health record data. Individuals with normal-tension glaucoma, a subtype of glaucoma where optic nerve damage occurs despite normal intraocular pressure (IOP), were excluded from the study to ensure a homogeneous cohort. Participants lacking age, sex, or genotype data were excluded as these individuals would not possess the expected covariates for the association analysis. Categories of genetically-determined ancestry in *All of Us* corresponded directly to categorical ancestry definitions used within gnomAD,^8^ the Human Genome Diversity Project,^9^ and 1000 Genomes:^10^ African/African American, Admixed American/Latino, East Asian, European, Middle Eastern, South Asian, and Other (meaning an individual’s predominant ancestry is < 50% of their total ancestral composition).

### Genotyping:

Details of genotyping procedures used by *All of Us* have been described previously^11^. We excluded samples with a variant call rate below 99% or fewer than five heterozygotes. No imputation was required in the *All of Us* research dataset, as data was generated from short-read whole genome sequencing across 403,916 individuals. Genomic analysis used the GRCh38 reference genome.^12^ We utilized the Allele Count Allele Frequency (ACAF) data in the *All of Us* Researcher Workbench. The ACAF threshold callset includes variants with a population-specific allele frequency (AF) greater than 1% or a population-specific allele count over 100 in any ancestral subpopulations. Quality control measures ensured genotype data reliability, including filtering out variants with an allele frequency less than 1% and Hardy-Weinberg Equilibrium outliers (p < 1×10^−10^)^13^. Logistic regression analysis was conducted separately for each autosome, considering covariates such as the top 11 genotyping principal components, sex, and age. Related individuals were excluded based on available relatedness data, including first-degree relatives (parents, siblings, cousins) to minimize confounding; specifically, one individual was retained randomly from each family.

Our analysis utilized *Hail*^14^ for scalable genomic data analysis, *Bokeh*^15^ for interactive visualization, *Pandas*^16^ for data manipulation, and *NumPy*^17^ for numerical computing.

### Genome-wide association study:

To account for potential population stratification amongst our study participants within ancestry categories, we projected everyone’s genotype by principal components using cohort-wide standardized genotypes.

We performed ancestry-specific GWAS analyses for each group of European, African, and Admixed American/Latino ancestries to explore genetic associations unique to each ancestry. For each population, we separately computed the top 11 genotyping PCs. To this end, we performed a logistic regression Wald test. Manhattan and quantile-quantile plots were generated to visualize the GWAS results and compute the genomic inflation factor, which could reveal unaccounted population stratification (lambda = 1.00). Genome-wide significant single nucleotide polymorphisms (SNPs) were identified at a threshold of p < 5×10^−8^ for each ancestry group and separately for the cross-ancestry meta-analysis.^18^

We defined a POAG-associated locus as a genomic region within ± 1 Mb of the lead variant. A locus was considered novel if it did not include any previously reported variants with a p-value < 5×10^−8^ in previous GWAS nor was in high linkage disequilibrium (r^2^ >0.1) with genome-wide significant POAG variants from previous GWAS.^18^ We employed the GWAS Catalog^19^ and Litvar^20^ databases to account for previous GWAS. If a genome-wide significant SNP landed in a protein-coding region of a gene, we also searched the GWAS Catalog to identify if this gene was associated with any potential comorbidities which may be physiologically connected with POAG.

### Fixed-effect meta-analysis and multi-ancestry GWAS:

We conducted a fixed-effect meta-analysis across three ancestry groups (European, African, and Admixed American/Latino) by integrating summary statistics from separate GWAS for each group. The remaining ancestry groups available in *All of Us* were not included in these analyses due to prohibitively small sample sizes.

We applied an inverse-variance-weighted fixed-effect meta-analysis to these ancestry-specific results, which enhanced our overall statistical power to identify POAG-associated variants. We estimated meta-analyzed effect sizes and standard errors for each variant and calculated p-values based on a normal distribution. This method integrated data from multiple ancestry groups, providing a comprehensive view of genetic associations that may be shared across populations.

### Study cohort characteristics:

We identified 4,305 cases of POAG and 369,949 controls without POAG. Among these participants, there were 2,302 cases of European ancestry, 1,339 cases of African ancestry, and 465 cases of Admixed American/Latino ancestry. (Table 1)

## Results

**Figure 1:**
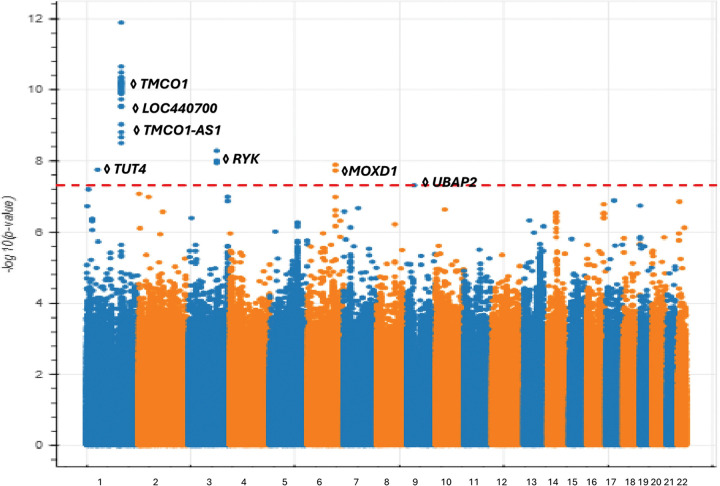
Manhattan plot demonstrating variants associated with POAG among All of Us participants of European ancestry.

European POAG GWAS identifies newly associated variants near genes with known roles in eye development and function:

The analysis of individuals of European ancestry (2,302 POAG cases, 213,774 controls) identified 52 genome-wide significant variants and five distinct loci associated with POAG, consistent with previous GWAS findings related to POAG and visual field loss.^21^ Notably, four of these loci were novel and have not been previously reported for POAG or glaucoma in general.(Fig. 1)( Table S1 (available at https://www.aaojournal.org))

On chromosome 1, we observed a significant number of associated variants near the *TMCO1* gene, which has been implicated in various disorders, including POAG, craniofacial dysmorphism, skeletal anomalies, and impaired intellectual development syndrome.^22^ *TMCO1* plays a crucial role in regulating intraocular pressure (IOP), a key factor in the development of POAG. Dysregulation of *TMCO1* may hinder the outflow of aqueous humor potentially resulting in elevated IOP levels.^23,24^ Additionally, on chromosome 1, we replicated the association near the pseudogene *LOC440700* and the *TMCO-AS1* gene which have both been previously reported in POAG GWAS.^25,26^

We identified several loci associated with POAG that have not been previously reported by existing GWAS. One such locus is centered at 52.5 Mb on chromosome 1 near the *TUT4* gene, which is responsible for uridylating miRNAs.^27^ This gene is related to glutathione peroxidase 7, where changes in enzyme activity may contribute to age-related macular degeneration (AMD).^28,29^ Furthermore, *TUT4* has been linked to height,^30^ with studies suggesting that individuals who are taller or have lower body mass index tend to have a smaller neuroretina rim area and a larger optic cup-to-disc area ratio.^31^

On chromosome 3, we discovered an associated locus consisting of intronic variants within the *RYK* gene. The *RYK* gene significantly influences eye development, particularly through its modulation of Wnt signaling pathways critical for eye organogenesis.^32^ Additionally, *RYK* has been shown to affect systolic and diastolic blood pressure,^33^ and numerous studies have demonstrated an association between blood pressure and POAG.^34–36^

We identified another POAG-associated locus centered on the promoter region of the *MOXD1* gene on chromosome 6 (at 132.2 Mb). *MOXD1* has been implicated in the progression of AMD^37^ and anemia.^38^ *MOXD1* is also known to affect tau protein levels, which may lead to modifications in neuronal injury associated with ocular hypertension.^39,40^ Lastly we identified a POAG-associated intronic variant on chromosome 9 which encodes *UBAP2*, a gene associated with the neurodegenerative disease amyotrophic lateral sclerosis^41^, in which astrocytes play a role in both ALS disease and in changes to the optic nerve head in glaucoma.^42^

### African ancestry GWAS identifies new loci not previously identified with European GWAS data:

**Figure 2:**
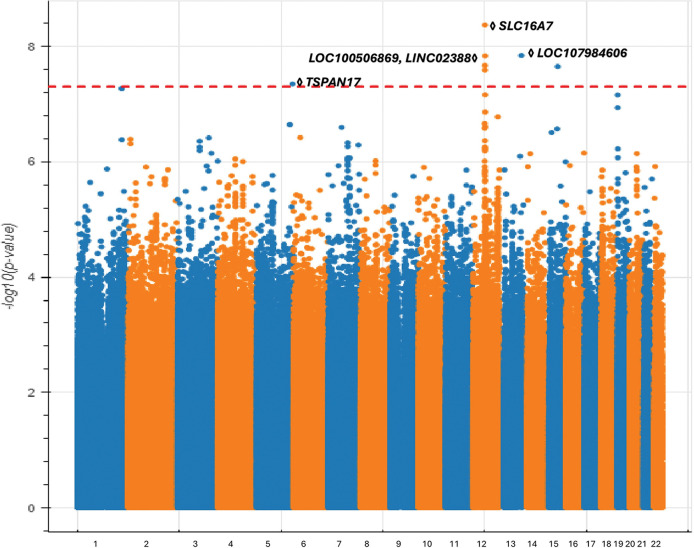
Manhattan plot demonstrating variants associated with POAG among All of Us participants of African ancestry.

During our investigation into the genetic factors contributing to POAG within the African ancestry group (1,339 POAG cases, 69,491 controls), we uncovered novel associations that highlight the intricate genetic complexity and remarkable diversity present in POAG genes in different populations. Our research identified seven genome-wide significant SNPs across five independent loci (as determined by distance and linkage disequilibrium) (Fig. 2, Table S1 (available at https://www.aaojournal.org)).

None of these associations have been previously identified in any GWAS related to POAG. Notably, this GWAS did not recapitulate the well-established *TMCO1* locus found in European POAG GWAS, suggesting that this gene may not play as critical a role in POAG pathogenesis in non-European individuals. This is supported by our analysis which found that the associated variants in the TMCO1 locus are specifically common in the European population, but rare in the African and Latino populations.

Here, we summarize these novel POAG-associated loci in order of genomic coordinates. First, we identified an associated locus on chromosome 5 centered on an intronic variant of the *TSPAN17* gene. The expression of *TSPAN17* in the neural tube and brain suggests a potential influence on neurological factors related to POAG.^43^ Second, on chromosome 12, we identified another associated locus centered on the *SLC16A7* gene, which has been implicated in age-related cataract and is expressed in retinal tissue.^44^ Notably, it has been shown that AMD and POAG exhibit a positive genetic correlation.^45^ Additionally, previous work indicates that *SLC16A7* may affect alcohol consumption^46^, which has been shown to increase the risk of glaucoma.^47^ Third, also on chromosome 12, but more than 1 Mb away, we identified a locus harboring two non-coding RNA genes: *LOC100506869* and *LINC02388*. The latter gene has been connected to cataract formation,^48^ which may contribute to primary angle-closure glaucoma due to a narrower drainage angle in the eye. While cataracts do not directly cause glaucoma, there are rare instances where cataracts can lead to elevated IOP and damage to the optic nerve.^49^

Lastly, we discovered an associated locus on chromosome 13 encoding the *LOC107984606* gene with no immediate connection to POAG pathogenesis, as well as an association on chromosome 15 centered on a nonfunctional variant which does not encode any gene.

**Figure 3:**
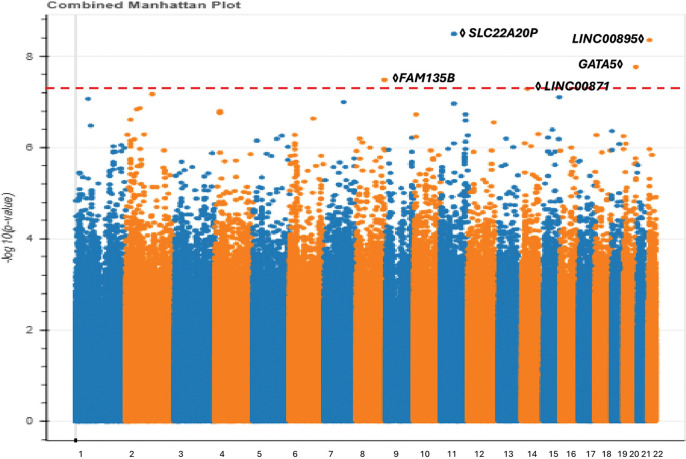
Manhattan plot demonstrating variants associated with POAG among All of Us participants of Admixed American/Latino ancestry.

All of Us cohort enables first POAG GWAS for individuals of Admixed American/Latino ancestry:

The modest sample size of the Admixed American/Latino population in our cohort (465 POAG cases, 67,875 controls) has enabled us to conduct an ancestry-specific GWAS for this demographic, whereas previous studies suffered from small sample size and thus were only powered to perform cross-ancestry meta-analysis.^25^ Our advance toward learning population-specific genetic susceptibility for POAG is critical as Latinos are approximately 5% more likely to be affected by POAG compared to other populations^50^. Our analysis led to the identification of five genome-wide significant variants, constituting independent loci. (Fig. 3, Table S1(available at https://www.aaojournal.org)).

First, we recapitulated an associated locus found by previous European POAG GWAS^25,51^ on chromosome 11 encoding the *SLC22A20P* gene. The genome-wide significant variants from the European GWAS ^25,51^ are in moderate linkage disequilibrium (r^2^ > 0.1) with our lead variant. The *SLC22A20P* gene has been shown to influence mean corpuscular hemoglobin levels,^52^ with higher levels correlating with a faster rate of retinal nerve fiber layer (RNFL) thinning.^52,53^ Second, we identified a genome-wide significant locus on chromosome 22, centered on an intergenic variant near encoding intergenic the non-coding RNA gene *LINC00895*. This locus is situated within ± 1 Mb of previously associated POAG variants^51^, although these variants were suggested to regulate different genes. Importantly, the variants identified in these previous studies exhibited low linkage disequilibrium (r^2^ < 0.1) with our lead variant, potentially suggesting that this finding may represent an independent mechanism. The *LINC00895* gene is known to affect platelet count, and lower platelet counts have been observed in individuals with POAG.^54^

We also identified several loci that have not previously been implicated in POAG. First, on chromosome 8, we identified an associated locus centered on an intronic variant of *FAM135B* which is associated with smoking behavior.^46^ Second, we identified a genome-wide significant intronic variant of *LINC00871* on chromosome 14. Expression of this gene is observed in the basal ganglia, particularly within the caudate and putamen nuclei. Prior GWAS has reported the association of this variant with Sjögren’s syndrome, which has implications for ocular dryness.^55^ Moreover, *LINC00871* has been associated with body mass index,^56^ suggesting potential pleiotropy affecting obesity and the development of POAG,^57^ as well as smoking status and initiation^58^ highlighting the impact of tobacco use on POAG.^59^

Third, on chromosome 20, we identified a POAG-associated variant 35 kb upstream of the *GATA5* gene, which is associated with AMD. Others have hypothesized that the mechanisms underlying the associations at the *GATA5* locus in neovascular AMD patients may be linked to retinoic acid signaling.^60^ Furthermore, *GATA5* has been shown to affect hematocrit levels,^61^ potentially contributing to increased IOP.^62^ Additionally, *GATA5* influences lung function,^63^ where reduced lung function has been associated with an increased risk of glaucoma.^64^

### Cross-ancestry GWAS meta-analysis:

In our analysis, we identified 56 genome-wide significant variants, 6 of which were not identified in ancestry-specific POAG GWAS. All but five of the genome-wide significant ancestry-specific GWAS variants were additionally found to be significant in the cross-ancestry meta-analysis. The exceptions mostly included variants identified in the Admixed American/Latino GWAS, which has a substantially smaller sample size and thus lower contribution to the cross-ancestry meta-analysis.

First, on chromosome 5, an intergenic variant was newly associated in the meta-analysis; the closest gene is ENSG00000286625 and is 10,000 Kb away. Second, we identified an intronic variant in the *SGCZ* gene on chromosome 8 that has previously been linked to BMI ^65^, reinforcing the possible role of metabolic pathways in glaucoma development. Third, we detected an intronic variant on chromosome 12, the *SLC16A7* gene influencing body weight and BMI^46^, suggesting a relationship between metabolic factors and POAG risk.^57^ Fourth, we identified one intronic variant on chromosome 16 in the *MAFTRR* and *LOC105371356* genes, both of which affect thyroid function, indicating a potential link between thyroid-related pathways and POAG susceptibility.^66^ Fifth, on chromosome 20, we identified an intronic variant in the *GGT7* gene, which is linked to chronic kidney disease (CKD), suggesting a potential association between glaucoma and CKD.^67^ Lastly, we identified a intron variant on chromosome 21,The gene *TRPM2*, which is a channel gene is associated with POAG, suggesting that TRPM2 may serve as a potential aqueous humor biomarker for glaucoma.^68,69^

## Discussion

Our study highlights the benefits of conducting genetic research in non-European populations. LD often poses a significant challenge in identifying causal variants in GWAS. However, an analysis of GWAS results from different ancestries with diverse LD structures can enhance the precision of causal variant identification. We performed this analysis for African ancestry, as well as for European and admixed American Latino populations. While previous GWAS have included non-European populations, such as those studied in the DIGS/ADAGES and NEIGHBORHOOD consortia^3^, a large proportion of prior research has focused on European ancestry groups.^3^ Based on our literature search, only one prior study has investigated POAG in admixed Latino populations^70^, highlighting the importance of exploring genetic contributions in these underrepresented groups. However, there remains a significant gap in our understanding of genetic risk factors in other admixed populations, despite the increasing incidence of POAG^71^ in these diverse communities. Additionally, our study identified novel loci and variants that have not been reported in earlier GWAS.

Our analysis revealed three new loci in European populations associated with genes *TUT4, RYK*, and *MOXD1*. Additionally, we identified five new loci from the African ancestry GWAS, as well as four novel loci in Admixed American/Latino ancestry. These results suggest that the genetic effects contributing to POAG may vary between populations, highlighting the importance of considering population-specific genetic architectures in complex traits. Given the significant differences in POAG prevalence across ancestries, it is likely that certain variants have ancestry-specific effects. Therefore, it is crucial to conduct ancestry-specific GWAS to uncover these unique genetic contributions.

In our study’s limitations, we acknowledge the relatively modest sample sizes for African, East Asian, Admixed American/Latino, and Middle Eastern populations, which may hinder the robustness of our GWAS findings in diverse ancestries. Additionally, the lack of data in *All of Us* on visual field measurements and IOP restricts our ability to assess the effects of novel variants or loci on these established factors that are known to be associated with POAG. In addition, phenotyping using EHR diagnostic codes has known limitations,^72^ but additional clinical data that may assist with more precise phenotyping, such as imaging, testing, and free-text notes, are currently not available in *All of Us*.

Our study marks a significant advancement in understanding the genetic aspects of POAG across diverse populations. The findings provide insights into the genetic architecture of POAG, emphasizing the importance of genetic diversity in understanding disease susceptibility. Addressing challenges through more inclusive research that includes clinical, environmental, and genetic data is essential for developing effective, personalized interventions. Ongoing research is needed to validate these findings and clarify the functional consequences of identified genetic variations, ultimately aiming to improve early detection and management of this sight-threatening condition.

## Figures and Tables

**Table 1.** Characteristics of *All of Us* participants with and without POAG with genotype data available for analysis.

	POAG Cases (N = 4,305)	Controls (N = 369,949)
Mean (Standard Deviation) Age	73.9 (10.6) years	56.17 (17.0) years
No. (%) Hispanic	572 (13.3% of cases)	70,329 (19% of controls)
No. (%) Male	2,195 (51% of cases)	146,223 (39.5% of controls)
No. (%) Female	2,110 (49% of cases)	223,726 (60.5% of controls)
No. (%) African ancestry	1,339 (31.1% of cases)	69,491 (18.8% of controls)
No. (%) Admixed American/Latino ancestry	465 (10.9% of cases)	67,875 (18.3% of controls)
No. (%) European ancestry	2,302 (53.5% of cases)	213,774 (57.8% of controls)
No. (%) Other ancestry	199 (4.5% of cases)	18,809 (5.1% of controls)

## Data Availability

This study was conducted using data from the All of Us Research Program. The data are available to registered and contolled researchers through the All of Us Researcher Workbench. Access to individual-level data is controlled and requires approval by the All of Us Research Program.

## Abbreviations and Acronyms

POAG: Primary open-angle glaucoma
GWAS: Genome-Wide Association Studies
IOP: Intraocular Pressure
AMD: Age-related Macular Degeneration
